# Internal Carotid Artery Pseudoaneurysm After Transsphenoidal Pituitary Tumor Resection: A Case Report

**DOI:** 10.7759/cureus.36539

**Published:** 2023-03-22

**Authors:** William Montagne, Nathan Lloyd, Emily Sagalow, Efrem Cox, Julian Hardman, Jee-Hong Kim

**Affiliations:** 1 Otolaryngology - Head and Neck Surgery, University of Nevada Las Vegas School of Medicine, Las Vegas, USA; 2 Neurosurgery, University of Nevada Las Vegas School of Medicine, Las Vegas, USA; 3 Neurointerventional Radiology, Desert Radiology, Las Vegas, USA

**Keywords:** internal carotid artery pseudoaneurysm, transsphenoidal surgical resection, endonasal endoscopic transsphenoidal surgery, pituitary sugery, chat gpt, management of severe epistaxis, internal carotid artery aneurysm, internal carotid artery cavernous aneurysm, severe epistaxis

## Abstract

Here, we present a case report on internal carotid artery pseudoaneurysm (ICAP) which highlights a rare but potentially life-threatening complication of transsphenoidal pituitary surgery. A 32-year-old male underwent resection of a pituitary tumor and developed a large cerebrospinal fluid (CSF) leak during surgery, which was reconstructed with a fat graft and nasoseptal flap. Postoperatively, he was recovering well and discharged without complications; however, eight days after surgery he returned with massive epistaxis and hematemesis. This was initially managed with endoscopic exploration, nasal packing, and transfusion of blood products. Imaging revealed a pseudoaneurysm on the right internal carotid artery. The patient was started on aspirin and clopidogrel, and a flow diverter stent was placed without complications. Our case emphasizes the importance of prompt recognition and management of vascular injuries such as an internal carotid pseudoaneurysm after transsphenoidal pituitary surgery to prevent catastrophic outcomes.

## Introduction

Transsphenoidal pituitary surgery is a common approach for the treatment of pituitary tumors. However, it carries the risk of internal carotid artery injury, which can result in serious complications. Internal carotid artery pseudoaneurysm (ICAP) is one of the rare but potentially fatal complications that can occur after transsphenoidal surgery [1]. It is a type of vascular injury that occurs due to damage to the arterial wall, resulting in the formation of a pulsatile sac filled with blood outside the arterial lumen. The pseudoaneurysm can compress adjacent structures, rupture, or thrombose, leading to stroke, hemorrhage or death.

## Case presentation

**Figure 1:**
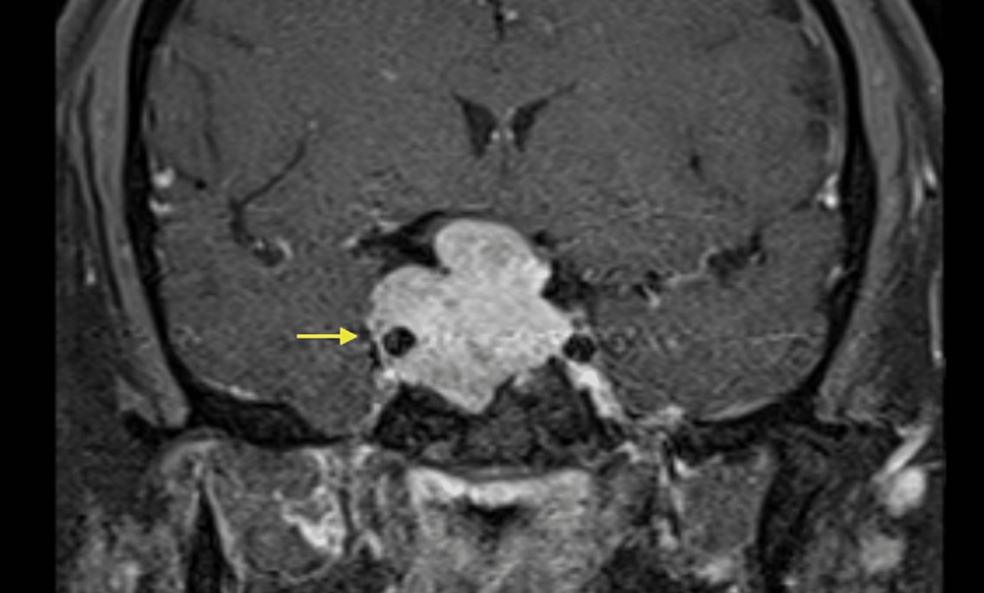
Pre-operative coronal MRI of pituitary mass T1 post-contrast fat saturation showing contrast enhancement of a large sellar/suprasellar mass with encasement of the cavernous portion of the right internal carotid artery (yellow arrow).

**Figure 2:**
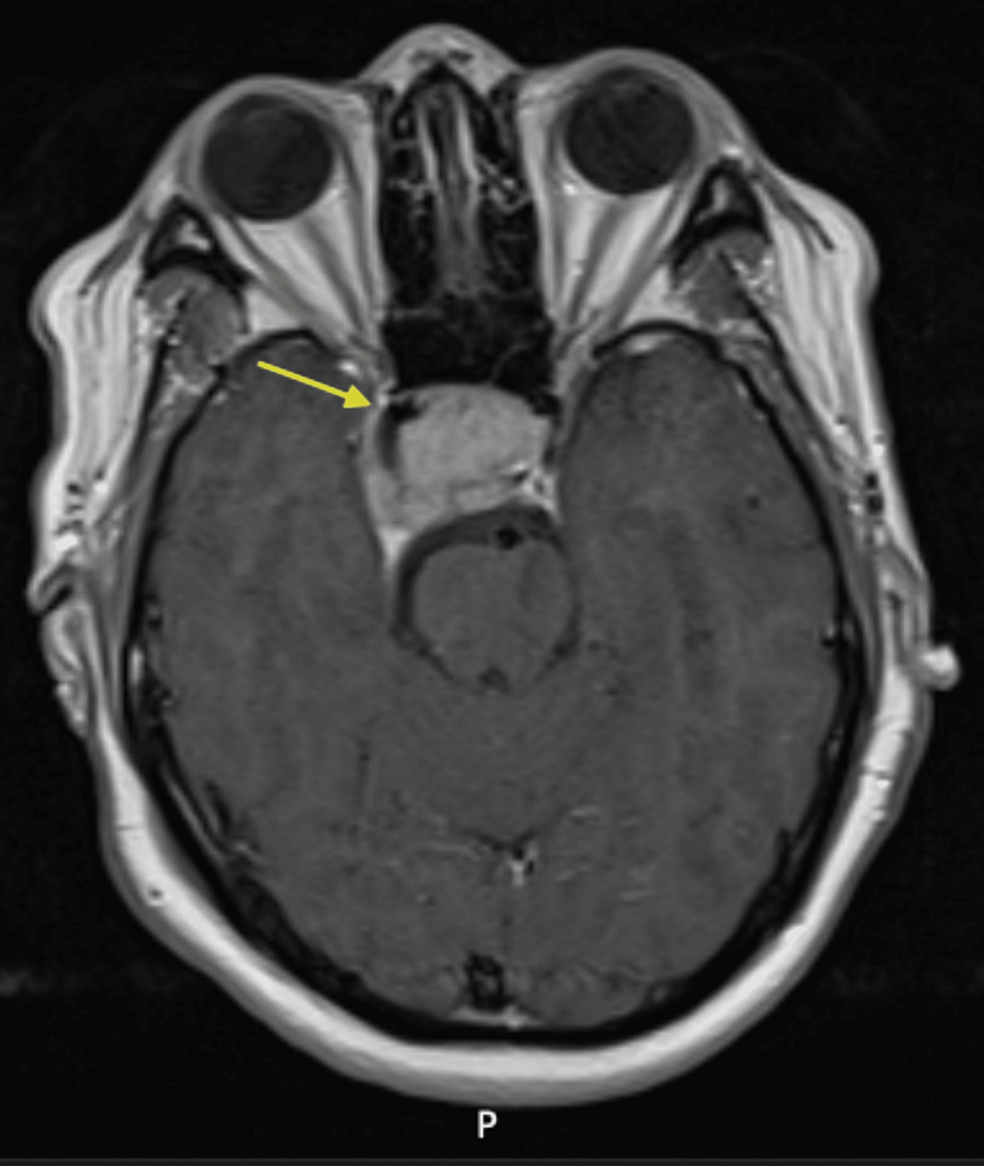
Axial MRI brain T1 SE post-contrast showing tumor around right internal carotid artery (yellow arrow).

**Figure 3:**
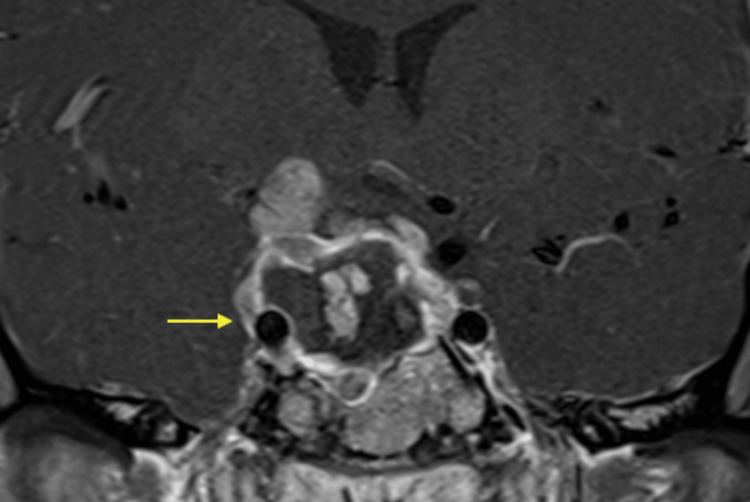
Postoperative coronal MRI of pituitary mass T1 post-contrast showing subtotal resection of previously described sellar/suprasellar mass. Residual enhancing tumor along the right aspect of the sella and right cavernous sinus (yellow arrow) extending to the suprasellar region. There is encasement of the right internal carotid artery.

**Figure 4:**
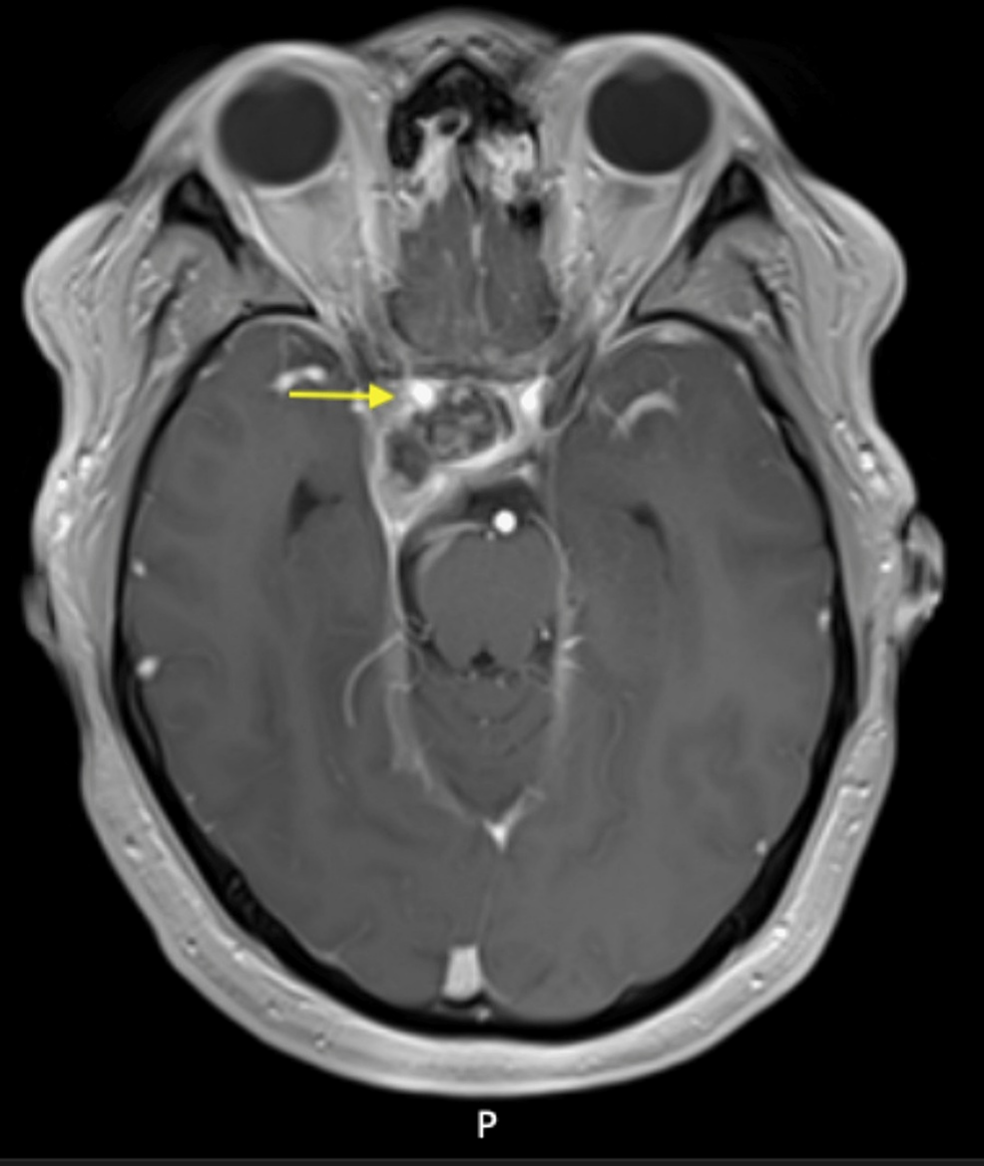
MRI brain axial T1 post-contrast showing right internal carotid artery (yellow arrow) with residual tumor and post-surgical changes.

A 32-year-old male presented to our hospital's emergency department with a 2-month history of vision changes and a known pituitary tumor. On examination, he had decreased peripheral vision, with the right side being worse than the left. The MRI pituitary and brain (Figures 1, 2) showed a large sellar and suprasellar mass measuring about 3.7 x 3.2 x 2.9 cm. The tumor was involved in the right cavernous sinus, encasing the right cavernous carotid artery, and causing a mass effect on the optic chiasm. The patient underwent transsphenoidal subtotal resection of the pituitary tumor. During the resection, there was a high-flow cerebrospinal fluid (CSF) leak, which was reconstructed with a fat graft and left nasoseptal flap. The nasal cavity was packed with dissolvable packing materials. Postoperatively, his vision improved and there were no signs of a CSF leak or bleeding. A postop MRI showed subtotal resection with a small residual tumor encasing the right internal carotid artery in the suprasellar region (Figures 3, 4). The remainder of his hospital stay was uncomplicated, and he was discharged POD 7.

On the evening of POD 8, he returned to the emergency department with large-volume nasal bleeding and hematemesis. The epistaxis was high flow, high volume, and the bleeding occurred around and through the packing, on the right side. A large amount of oxymetazoline was used on the packing in both nostrils, and dissolvable packing was placed by the on-call otolaryngology team. The patient was noted to be pale and found to be tachycardic to 120s. Due to the large volume of epistaxis, the decision was made to take the patient emergently to the operating room (OR) to explore the possibility of arterial bleeding. Type & screen and three units of blood products were held for the OR. While being transported to the OR, he had right-sided epistaxis around the packing again. He was intubated through rapid sequence induction and the packing was endoscopically removed from the right side of the nasal cavity. During the nasal cavity exploration, a large volume of blood was noted to pool from the right sphenoid sinus above the CSF leak repair site. Packing with gauze was necessary to control the bleeding. The patient was transfused with red blood cells while he was taken emergently to interventional radiology.

**Figure 5:**
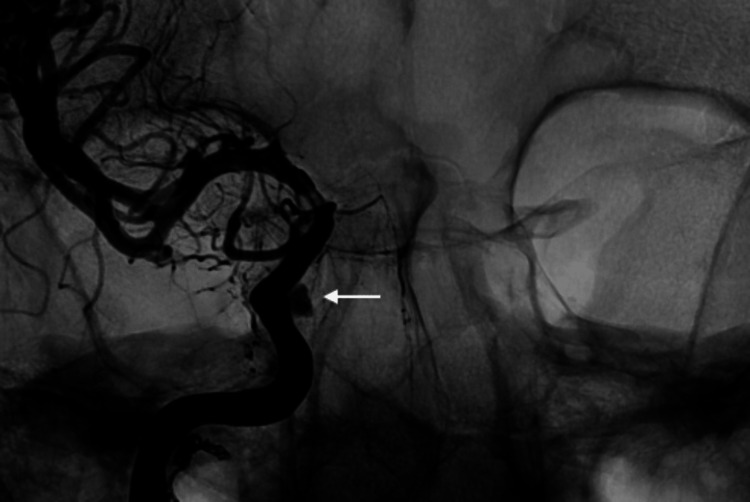
Anterior posterior view of right internal carotid artery pseudoaneurysm (white arrow) around the level of the cavernous carotid.

**Figure 6:**
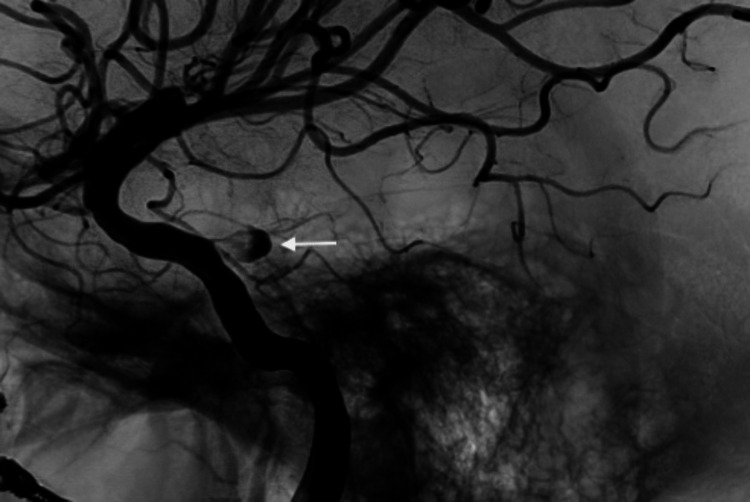
Lateral view of the internal carotid artery pseudoaneurysm (white arrow).

**Figure 7:**
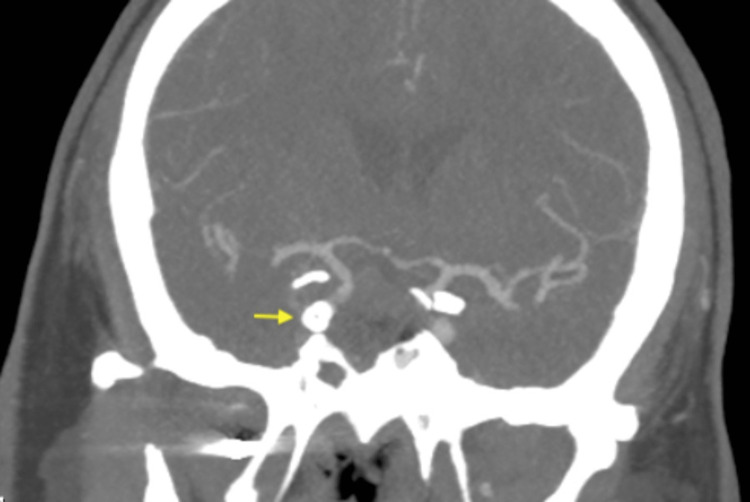
CTA coronal MIP image showing the right internal carotid artery flow diverter (yellow arrow) with filling of the distal internal carotid artery.

**Figure 8:**
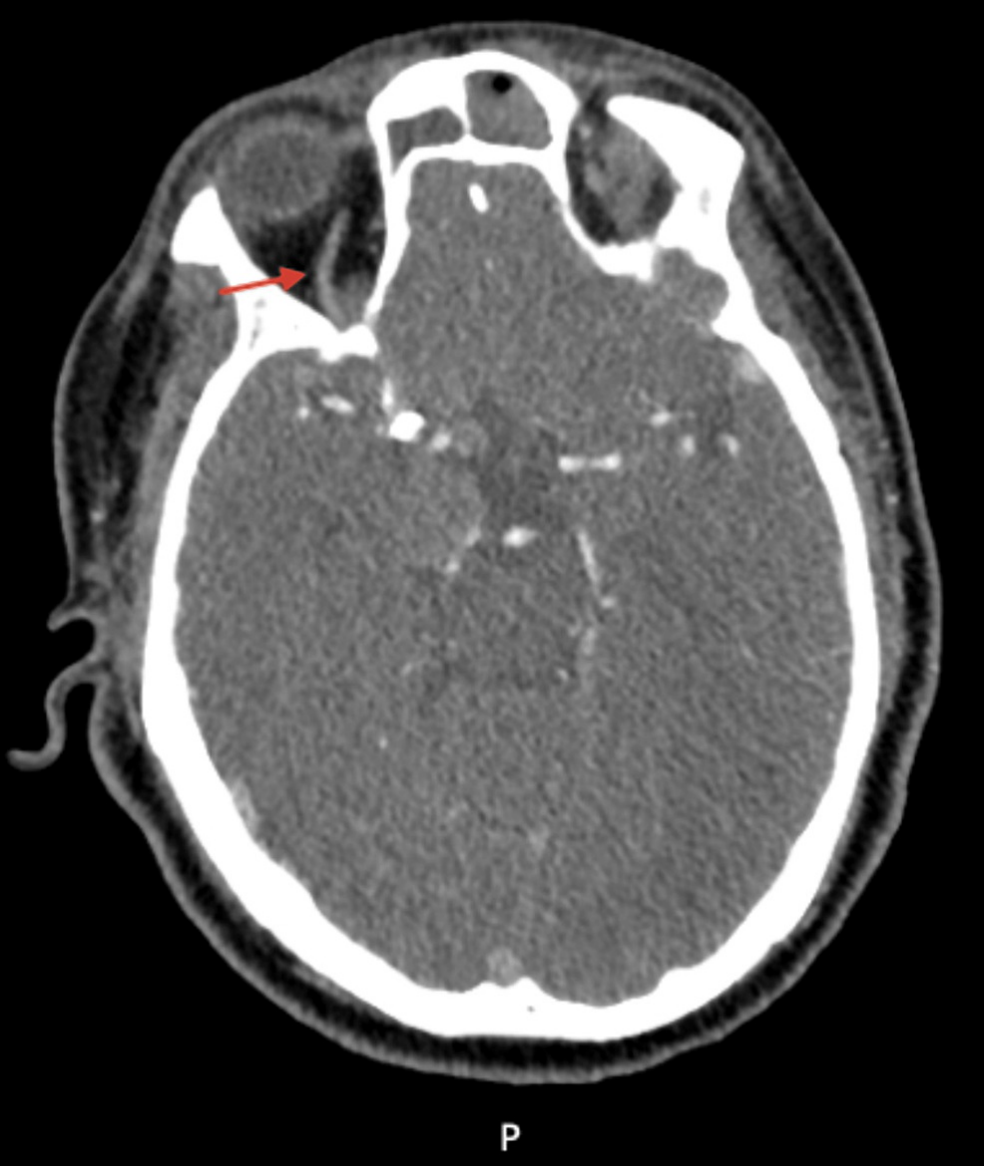
CT angiogram of the head after flow diverter placement showing a patent right ophthalmic artery (red arrow)

A carotid artery angiogram was performed which revealed a pseudoaneurysm on the right side of the internal carotid artery around the level of the cavernous sinus (Figures 5, 6). The patient was given a loading dose of Clopidogrel 300 mg and Aspirin 650 mg prior to successful placement of the right internal carotid artery flow diverter device (Pipeline Flex, Medtronic, Minneapolis, MN, USA). On the third day after flow diverter device placement, a CT angiogram of the head was performed (Figure 7), which revealed a patent flow diverter device. The CT angiogram of the head also showed a patent right ophthalmic artery (Figure 8). Later that day, the patient underwent nasal endoscopy and removal of the packing by the otolaryngology team in the operating room. No carotid bleeding or CSF leak was observed. Following surgery, the patient remained neurologically intact without further bleeding episodes when last seen during one month follow-up.

## Discussion

Transsphenoidal pituitary surgery is a common procedure used to treat pituitary tumors located at the base of the brain. Despite being a minimally invasive approach, it carries the risk of complications, including vascular injury. Here we presented a case of ICAP after transsphenoidal surgery. This is a rare, but potentially fatal vascular complication. ICAP occurs when there is a disruption of the arterial wall that leads to a sac-like structure filled with blood outside the arterial wall. In this paper, we will discuss the management and treatment options for ICAP after transsphenoidal pituitary surgery, including endovascular and surgical techniques.

The reported incidence of ICAP in skull base surgery ranges from 0.4% to 7.4% [1-4]. The time of diagnosis of ICAP is usually reported between two days to 10 years postoperatively. The risk factors for developing ICAP after transsphenoidal surgery include a history of hypertension, previous radiation therapy, tumor invasion of the cavernous sinus, prior treatment with bromocriptine, acromegaly, and extensive surgical resection of the sellar region [2,5]. The clinical presentation of ICAP can vary from an asymptomatic finding on imaging to life-threatening complications such as subarachnoid hemorrhage, carotid-cavernous fistula, or even death [6]. Mortality rates from rupture of ICAP have been reported up to 50% [1,2]. When ICAP presents with large epistaxis, attempting to control it with cauterization or hemostatic material alone may worsen or fail to control the situation. Control of carotid bleeding is usually best done with multiple cotton pads or gauze [7]. Initial stabilization of bleeding should always be the first priority. Once the bleeding is stabilized, one must volume resuscitate the patient to prevent hemorrhagic shock or ischemic stroke and secure their airways. 

Prompt diagnosis and management are essential to prevent catastrophic outcomes. The gold standard for the diagnosis of ICAP is digital subtraction angiography (DSA) [1]. In hemodynamically stable patients, a CT angiogram can be used for the diagnosis of ICAP. When considering an internal carotid artery injury there are a variety of potential treatment options including endovascular and surgical [8]. Endovascular management options include permanent occlusion, coil embolization, stenting, flow diverter stent, or pipeline embolization device which have all been reported as safe and effective treatments for ICAP [3,4,9,10]. However, surgical management with arterial ligation or revascularization may be necessary in some cases [1,7]. When considering embolizing or sacrificing the internal carotid artery, physicians must consider collateral circulation in the Circle of Willis. Lack of appropriate cerebral blood flow or collateral circulation could lead to unnecessary morbidity from neurologic deficits. Although not available at our hospital, a hybrid operating room that has the ability to do DSA may help with both the diagnosis and treatment of ICAP. Once the ICAP has been treated removal of any nasal packing should be done in the OR or interventional radiology suite as bleeding can occur after removal. After treatment of ICAP, it is important to get a follow-up angiogram to evaluate any abnormalities of the internal carotid.

The use of flow diverters provides an excellent option for the endovascular treatment of intracranial pseudoaneurysms; however, their use does not come without risks. Complication rates have been reported around 17% with permanent morbidity rates and mortality rates of 3.7% and 2.8%, respectively [11]. Rates of rebleeding and ophthalmic artery occlusion have been reported at around 3% each [11,12]. Other possible complications include ipsilateral stroke, stent migration/occlusion, thromboembolism, need for additional endovascular intervention, epistaxis, groin hemorrhage, gastrointestinal bleeding, and death. These risks can be decreased with proper surgical technique, antiplatelet therapy, and close monitoring in the postoperative period.

It is fortunate that the authors were in a situation where gauze was able to control the bleeding from the ICAP. In other situations, this may not be the case and more rapid and aggressive treatments may be needed. We also were fortunate to have a neuro-interventional radiologist readily available, which is not the case at all institutions. It is important to treat each ICAP on a case-by-case basis and also consider what treatment pathways are available within your hospital system. 

**Figure 9:**
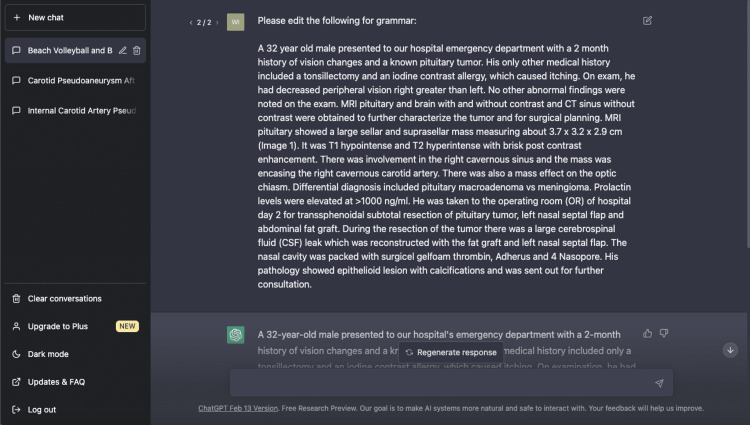
Experience with ChatGPT assist with editing.

**Figure 10:**
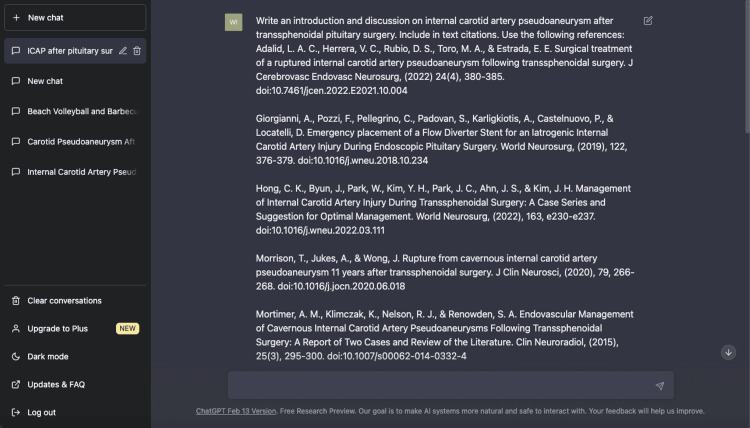
Experience with ChatGPT to assist with writing.

Figures 9, 10 in the appendices show our experience using ChatGPT.

## Conclusions

Overall, ICAP is a rare but potentially fatal complication of transsphenoidal pituitary surgery. The risk factors for ICAP should be carefully considered preoperatively, and prompt diagnosis and management are essential to prevent catastrophic outcomes. The initial focus in a bleeding patient should be control of bleeding which can best be accomplished by packing with non-absorbable materials. Endovascular management with balloon occlusion, embolization, flow diverter stent, or pipeline embolization device has been reported as a safe and effective treatment for ICAP, and surgical management with arterial ligation or revascularization may be necessary in some cases. Following treatment, packing should be removed in the OR or interventional radiology suite as bleeding can occur. A multidisciplinary approach involving neurosurgery, interventional neuroradiology, otolaryngology, and vascular surgery is essential for the optimal management of ICAP.
